# Age-Related Intraneuronal Elevation of αII-Spectrin Breakdown Product SBDP120 in Rodent Forebrain Accelerates in 3×Tg-AD Mice

**DOI:** 10.1371/journal.pone.0037599

**Published:** 2012-06-18

**Authors:** Yan Cai, Hai-Xia Zhu, Jian-Ming Li, Xue-Gang Luo, Peter R. Patrylo, Gregory M. Rose, Jackson Streeter, Ron Hayes, Kevin K. W. Wang, Xiao-Xin Yan, Andreas Jeromin

**Affiliations:** 1 Department of Anatomy and Neurobiology, Central South University Xiangya Medical School, Changsha, Hunan, China; 2 Department of Neurology, The Third Xiangya Hospital, Changsha, Hunan, China; 3 Neuroscience Research Center, Changsha Medical University, Changsha, Hunan, China; 4 Departments of Anatomy & Physiology, Southern Illinois University School of Medicine, Carbondale, Illinois, United States of America; 5 Center for Integrated Research in Cognitive and Neural Sciences, Southern Illinois University School of Medicine, Carbondale, Illinois, United States of America; 6 Banyan Biomarkers, Alachua, Florida, United States of America; Univ. Kentucky, United States of America

## Abstract

Spectrins line the intracellular surface of plasmalemma and play a critical role in supporting cytoskeletal stability and flexibility. Spectrins can be proteolytically degraded by calpains and caspases, yielding breakdown products (SBDPs) of various molecular sizes, with SBDP120 being largely derived from caspase-3 cleavage. SBDPs are putative biomarkers for traumatic brain injury. The levels of SBDPs also elevate in the brain during aging and perhaps in Alzheimer’s disease (AD), although the cellular basis for this change is currently unclear. Here we examined age-related SBDP120 alteration in forebrain neurons in rats and in the triple transgenic model of AD (3×Tg-AD) relative to non-transgenic controls. SBDP120 immunoreactivity (IR) was found in cortical neuronal somata in aged rats, and was prominent in the proximal dendrites of the olfactory bulb mitral cells. Western blot and densitometric analyses in wild-type mice revealed an age-related elevation of intraneuronal SBDP120 in the forebrain which was more robust in their 3×Tg-AD counterparts. The intraneuronal SBDP120 occurrence was not spatiotemporally correlated with transgenic amyloid precursor protein (APP) expression, β-amyloid plaque development, or phosphorylated tau expression over various forebrain regions or lamina. No microscopically detectable *in situ* activated caspase-3 was found in the nuclei of SBDP120-containing neurons. The present study demonstrates the age-dependent intraneuronal presence of an αII-spectrin cleavage fragment in mammalian forebrain which is exacerbated in a transgenic model of AD. This novel neuronal alteration indicates that impairments in membrane protein metabolism, possibly due to neuronal calcium mishandling and/or enhancement of calcium sensitive proteolysis, occur during aging and in transgenic AD mice.

## Introduction

Spectrins were first discovered in red blood cells and have since been identified to be ubiquitously expressed, including in neurons and glia [1],[2],[3],[4],[5],[6],[7],[8]. The spectrin family of proteins includes a group of closely related gene products that assemble as tetramers of α and β subunits to form a pentagonal or hexagonal submembranous meshworks by cross-linking with other proteins such as actin, protein 4.1 and ankyrin. Spectrin filaments are essential for cells to maintain stability of the membrane bilayer and cytoskeleton [3],[6]. These proteins also participate in assembly of specialized membrane domains during dynamic membrane remodeling events, such as cell migration, neuritic outgrowth and synaptogenesis, therefore allowing membranous and cytoskeletal flexibility that may be vital for neuronal and synaptic plasticity [9],[10],[11],[12],[13],[14],[15],[16],[17]. The spectrin gene family has expanded during evolution: one α and two β genes code spectrin subunits in invertebrates, whereas two α spectrins (αI and αII) and five β spectrins (βI to V) code the spectrin family proteins in vertebrates, including human [6].

Spectrins can be degraded proteolytically by enzymes, including calpains and caspases. During apoptotic and necrotic cell death, calpain-specific cleavage of αII-spectrin yields 145 kDa and 150 kDa breakdown products (SBDPs), namely SBDP145 and SBDP150. Caspase-3 mediated αII-spectrin proteolysis results in the release of SBDP120 and SBDP150 fragments [18],[19]. Thus, specific antibodies detecting these breakdown products can help differentiate cell death models [20],[21],[22],[23],[24]. Studies have shown SBDP elevation in the brain under acute and subacute conditions associated with neuronal stress, injury and death, including traumatic brain injury [21],[24], chemical neurotoxicity [25], hypoxia [26] and ischemia [27].

Alterations in spectrin metabolism appear to occur in the body as well as the brain during normal aging as well as age-related chronic neurodegenerative diseases [28]–[34]. Specifically, spectrin cleavage fragments (150 kDa) and abnormal spectrin immunoreactivity have been shown in the brains of Alzheimer’s disease (AD) patients [35]. One study also demonstrated accumulation of SBDP120 in cortical pyramidal neurons in AD, but not age-matched control, brains [36]. More recently, levels of SBDP145 and SBDP150 were found to be elevated in both CSF and brain in AD patients, as well as in transgenic models of AD [37],[38], suggesting the potential use of SBDPs as novel biomarkers for this disease [34].

Transgenic rodent models may be useful to explore the cellular and molecular basis underlying SBDP alterations in AD. Currently, little is known about the time course and cellular localization of SDBP appearance in the aged brain. Further, it is unclear if SDBPs are associated in any manner with the principal AD lesions, i.e., α-amyloid plaques or tau pathology. Using a novel and specific antibody, we identified the presence of SBDP120 in forebrain neurons beginning around mid-age in wild-type mice. In the triple transgenic mouse model of AD (3×Tg-AD) [39], SBDP120 expression occurred earlier and was more robust. The age-related SBDP120 intraneuronal labeling in 3×Tg-AD mice did not correlate anatomically or temporally with the development of extracellular amyloid plaques or tau pathology.

## Materials and Methods

### Ethics Statement

Experimental use of rats and mice in the present study was in accordance with the National Institutes of Health Guide for the Care and Use of Laboratory Animals. All experimental procedures used the present study were approved by the Ethics Committee for Animal Use at Central South University and by the Animal Care and Use Committee of Southern Illinois University at Carbondale.

### Animals and Tissue Preparation

Sprague-Dawley rats at 16 (n = 4) and 24–26 (n = 5) months of age, and in-house bred 3×Tg-AD mice and non-Tg controls at 6, 12, 18 and 24 months of age (n = 8/age point/genotype) were examined in the present study [40]. Animals were perfused transcardially under terminal anesthesia (sodium pentobarbital, 100 mg/kg, i.p.) with cold 0.01 M phosphate-buffered saline (pH 7.4, PBS). Brains were then quickly removed from the skull and bisected along the midline. One hemi-brain was snap-frozen with liquid nitrogen and stored at −70°C for western blot analysis. The other hemi-brain was fixed by immersion in 4% paraformaldehyde in 0.01 M phosphate buffer for neuroanatomical studies. After fixation for two days, the tissue was transferred into 30% sucrose in 0.01 M phosphate buffer and stored at 4°C until it sank. The brains were then cut into consecutive sets of coronal or sagittal sections at either 30 micron- (12 sets, stored in cryoprotectant at −20°C) or 6 micron- (12 sets, thaw-mounted on slides, stored at −20°C) thickness using a cryostat (Microm HM525, Germany). Markers (small cortical cuts or needle holes) were made before or during brain sectioning and were recorded for individual brains of different age groups. The 30 micron-thick sections were used for immunodetection of SBDP120, 6E10, phosphorylated-tau (p-Tau) or active caspase-3 with the avidin-biotin complex (ABC) method. In order to eliminate potential inter-experimental variability in immunolabeling, brain sections from a group of animals at different ages were batch-processed throughout the immunohistochemical procedure.

### Primary Antibodies

The rabbit anti-SBDP120 antibody was generated using a synthetic peptide (SVEALIKKHE) corresponding to the caspase-3 cleavage sequence (a.a.1478–1488) of αII spectrin. The final working dilution was set at 1∶400 by pilot antibody titration immunohistochemical tests. The specificity of this rabbit SBDP120 antibody was characterized in the current study by preabsorption of the primary antibody with the immunogenic peptide as well as by omission of the primary and secondary antibodies during immunohistochemistry procedures. Other primary antibodies employed were a mouse anti-β-amyloid peptide (Aβ) monoclonal antibody 6E10 (#39320, Signet, 1∶4000), a mouse anti-human phosphorylated tau (p-Ser396/Ser404) monoclonal antibody (PHF1, courtesy of Dr. P Davis, 1∶4000) and rabbit anti-active caspase-3 (#559565, BD Biosciences, 1∶2000) [40],[41].

### Immunohistochemistry

Sections (30 microns thick) were treated with 1% H_2_O_2_ in PBS for 30 minutes, then pre-incubated in 5% normal horse serum with 0.1% Triton X-100 for 1 hour. Sections were subsequently incubated overnight at 4°C with primary antibodies at pre-optimized concentrations in PBS containing 5% normal horse serum and 0.1% Triton X-100. Following incubation with a primary antibody, the sections were reacted with a biotinylated pan-specific secondary antibody at 1∶400 for 1 hour, and then in freshly prepared avidin-biotin complex (ABC) solution (1∶400) (Vector Laboratories, Burlingame, CA) for another hour. Immunoreactivity was visualized using 0.003% H_2_O_2_ and 0.05% diaminobenzidine (DAB). All incubations were followed by three 10 minute washes; after DAB development the sections were dehydrated and mounted. Some immunolabeled sections were counterstained with toluidine blue before mounting. For the purpose of densitometry (to define the levels of non-specific reactivity), brain-level matched sections were processed along with the experimental sections, but without the exposure to the primary antibody.

### Western Blot

Frontal cortical blocks (containing the anterior ∼¼ of a hemisphere and olfactory bulbs) were separated from frozen hemi-brains, weighed and homogenized by sonication in T-PER buffer (10×w/v) (Pierce, Rockford, IL) containing protease inhibitors (Roche, Indianapolis, IN). After centrifugation at 15,000×*g* at 4°C for 10 minutes, the supernatants were collected, followed by determination of protein concentration by DC protein assay (Bio-Rad Laboratories, Hercules, CA). Samples containing a total of 50 µg protein were run on a 4–20% SDS-PAGE gel (Bio-Rad Laboratories). The polypeptides were then electrotransferred into Trans-Blot® pure nitrocellulose membranes (Bio-Rad Laboratories) and immunoblotted for SBDP120 (1∶1000) or α-actin (1∶5000, Millipore, #04-1116). Immunoblotted protein products were visualized using HRP-conjugated goat anti-rabbit IgG (1∶20000, Bio-Rad Laboratories) and the ECL Plus™ Western Blotting Detection kit (GE Healthcare Life Sci., Piscataway, NJ). Immunoblot images were analyzed using a SI PhosphorImager (Model 475, Molecular Dynamics, GE Healthcare).

### Imaging, Data Analysis, Statistic Testing

Sections were examined on an Olympus (BX53) fluorescent microscope equipped with a digital camera and image analysis system (cellSens Standard, Olympus). Images (2070 x 1548 pixels) were taken using 4X to 40X objective lens (with a 10X ocular lens). For densitometric analysis, images were captured with the 20X objective lens using the same exposure setting for all brain groups. Optical densities over layers II-VI of the frontal cortex, and the mitral cell layer (MCL) together with the external plexiform layer (EPL) of the olfactory bulb, were measured with OptiQuant analysis software using the irregular-connecting sampling tool (Packard Instruments, Meriden, CT). For a given brain, optical densities were obtained from the three most medial sagittal sections (∼300 µm apart from each other), followed by a calculation of the average density. Row data were imported into and further processed with Microsoft Excel. Specific optical densities were calculated by subtracting a mean background density from total measured densities in a given area. The mean background density was the average of nonspecific reactivity readings obtained over the cortex and olfactory bulb in sections processed without the primary antibody. Means of specific optical density (o.d.) or normalized levels of specific o.d. between/among comparing groups were analyzed statistically using one-way ANOVA with Bonferroni posttests (Prism GraphPad 4.1, San Diego, CA). The minimal significance level was set at p<0.05. Figure panels were assembled using CorelDRAW 10.0, and then converted into TIFF format.

## Results

### Characterization of SBDP120 Immunoreactivity in Aged Rat Forebrain

**Figure 1:**
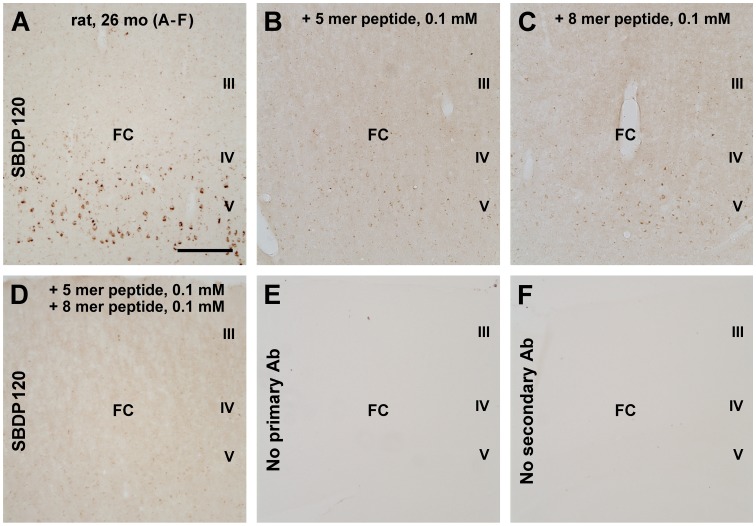
Immunohistochemical characterization of a rabbit antibody to 120 kDa αI-spectrin breakdown product (SBDP120) in aged rat frontal cortex (FC). Panel A shows SBDP120 labeling in a subpopulation of layer V (5) cortical neurons. This cellular labeling was largely blocked by the 5-mer (B) or the 8-mer (C) immunogenic peptide (at 0.1 mM concentration) used for antibody genesis. When both peptide forms were included in the primary antibody incubation buffer, specific labeling in the cortex was completely eliminated (D). No labeling was seen in the absence of the primary (E) or secondary (F) antibody (Ab). Scale bar = 400 µm in A, also applying to B-F.

SBDP120 antibody labeling was examined in brain sections from 16 and 24–26 month-old rats because our pilot study did not detect labeling in the cerebral cortex of 4- and 8-month old animals. SBDP120 IR in the frontal cortex of 24–26 month-old rats, most prominently in layer V (Fig. 1A). To determine the specificity of the labeling, adjacent sections were stained in parallel with and without the inclusion of the immunogenic peptide in the primary antibody incubation buffer. The SBDP120 labeling was largely absent in sections processed in the presence of either the 5-mer or 8-mer synthetic immunogenic peptide at 0.1 mM concentration (Fig. 1B, C). When both forms of peptide (0.1 mM) were added, the specific IR was completely eliminated (Fig. 1D). No cellular labeling existed in parallel batches of sections that were processed by excluding either the primary (Fig. 1E) or the secondary (Fig. 1F) antibody.

**Figure 2:**
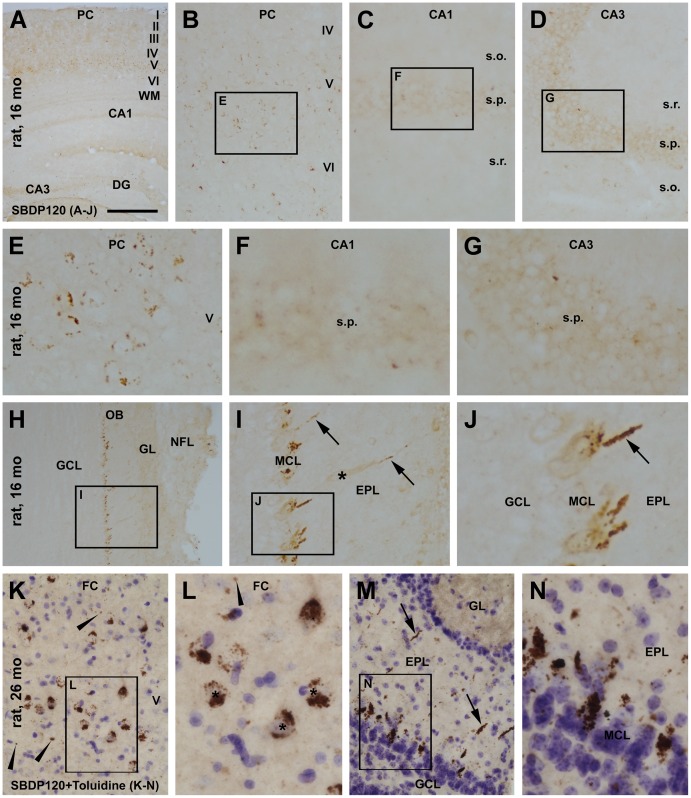
Representative images showing SBDP120 immunoreactivity in the cerebral cortex, hippocampal formation and olfactory bulb in 16- and 26-month (mo) old rats. Panel A is a low magnification image over the parietal cortex and underlying hippocampal formation, with subregions of this figure enlarged sequentially as panels B to G. Small dot- or granule-like immunoreactive elements were seen in deep cortical layers, especially layer V (B). These elements appear to group as perikaryal profiles around the cell boundary at high magnification (E). Much granular labeling was seen in stratum pyramidale (s.p.) of CA1 and CA3 (C, D, F, and G). In the olfactory bulb, a row of labeled profiles was visible at low magnification around the periphery (H), appearing as strong immunoreactive profiles around the mitral cell layer at high magnification (I, J). The labeling consisted of aggregated granules (arrows) located at the apical dendrites and the somatodendritic junction of the mitral cells. Asterisk in “I” indicates a displaced mitral cell or tufted cell. Panels K-N are high-magnification images of SBDP120 labeling in sections counterstained with toluidine blue from a 26 month-old rat. Relative to (E), larger and more densely labeled aggregated SBDP120 reactive elements were present in the cortex, some clearly appearing as pyramidal cellular profiles. Small dot-like and isolated profiles (arrowheads) were also seen (K, L). In the olfactory bulb (M, N), heavily labeled profiles in oval or fusiform shape were present around the mitral cell layer (MCL). Labeled segments were also seen in small numbers in the external plexiform layer (EPL) (arrows in M). Diffuse labeling seen in the glomeruli appeared to be somewhat increased in 26 (M) relative to 16 (H) month-old animals. Roman numerals denote cortical layers; WM: white matter; FC: frontal cortex; PC: parietal cortex; DG: dentate gyrus; OB: olfactory bulb; GL: glomerular layer; GCL: granule cell layer; MCL: mitral cell layer; EPL: external plexiform layer; ML: molecular layer; s.r.: stratum radiatum; s.l.m.: stratum lucunosum-moleculare. Scale bar  = 1 mm in A; equivalent to 300 µm for B-D, H, K, M; 100 µm for E-G, I, L, and 50 µm for J, N.

The pattern of SBDP120 IR in the cerebral cortex, hippocampal formation and olfactory bulb was compared in 16 and 24–26 month-old rats (Fig. 2). In 16 month-old aged rats (Fig. 2A–J) a small number of labeled profiles were seen in layer V across the cortex along the rostrocaudal dimension (Fig. 2A, B). At higher magnification these profiles appeared as small dot- or granule-like elements that were seemingly arranged along the border of neuron-like somatic profiles (Fig. 2B, E), rather than in the nucleus or cytoplasm. No apparent SBDP120 IR was visible in the hippocampal formation at low magnification (Fig. 2A), although a few labeled granular elements were detectable in stratum pyramidale (s.p.) of CA1 and CA3 at higher magnification (Fig. 2C, D, F, G). Fairly distinct SBDP120 IR occurred selectively in the outer areas of the olfactory bulb, arranged as a row of irregularly-shaped elements approximately along the mitral cell layer (Fig. 2H). At higher magnifications, the labeling at this location also appeared as granules, tending to aggregate at the peripheral pole and/or in the apical dendrites of a subpopulation of mitral cells (Fig. 2I, J), including a few displaced mitral cells or tufted cells in the external plexiform layer (Fig. 2I, marked with an asterisk).

Overall, the regional and laminar pattern of SBDP120 IR in the forebrain of 24–26 month-old rats (Figs. 1A, 2K–N) was comparable to that seen in 16 month-old animals. However, the amount and intensity of labeling in the cortex and olfactory bulb increased in the brains of the older rats (compare Figs. 1A and 2B). The shape, size and intensity of the labeled profiles varied considerably. Relatively larger or more heavily stained elements appeared to arrange as perikaryal profiles, as was clearly seen in sections counterstained with toluidine blue (Fig. 2K, L). The immunolabeling product appeared to occur intracellularly in addition to being arranged around the cell border (Figs. 1A, 2L). The intracellular labeling was granular, and in many cases condensed unevenly in pyramidal-like neurons (Fig. 2K, L). The apical dendrites of some pyramidal somata were sometimes also lightly labeled. Isolated immunoreactive elements were seen distant from toluidine-stained cell nuclei, suggestive of neuronal process labeling (Fig. 2K, L, arrowheads). In the olfactory bulb, heavily labeled profiles resided around, or slightly peripheral, to the mitral cell layer (Fig. 2M). These were composed of aggregated granules grouped in fusiform, rod and irregular shapes (Fig. 2N). In addition, some radially-oriented elements were scattered across the external plexiform layer (Fig. 1M), with fewer and smaller immunoreactive profiles also present near the glomeruli (Fig. 1M). An increased diffuse labeling was noticeable in the olfactory glomeruli in 24–26 month-old, relative to 16 month-old, rats (compare Figs. 2H and 2M).

### SBDP120 Immunoreactivity in Wild Type and 3×Tg-AD Mouse Forebrain

**Figure 3:**
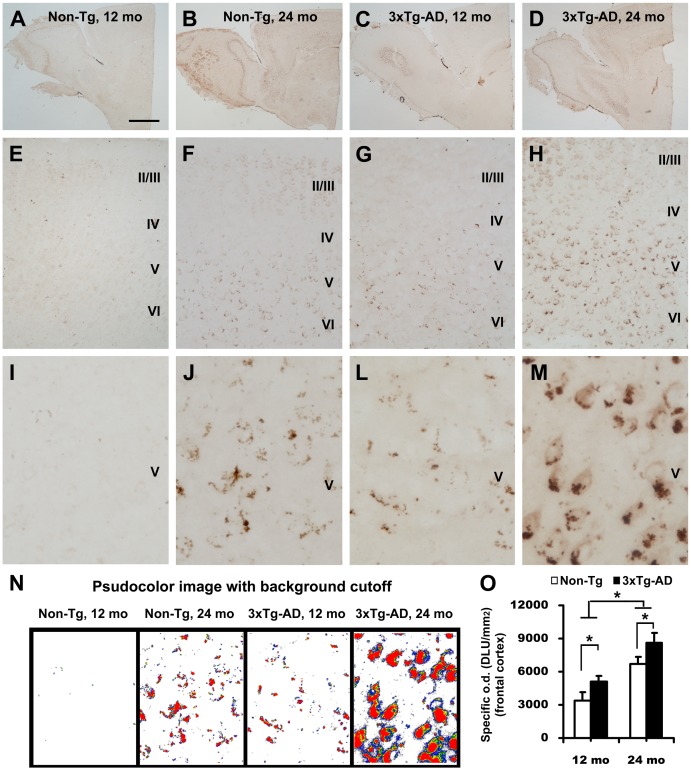
Images and densitometry showing increased SBDP120 immunolabeling in the frontal cortex of 3 ×**Tg-AD mice relative to wild type (Non-Tg) cohorts at 12 and 24 month of age.** Panels A–D are low magnification images illustrating the overall pattern of labeling in the forebrain. Labeling in the frontal cortical area was progressively enhanced with aging and transgenic status (E–M). Labeled profiles were hard to detect in the cortex in 12-month old non-Tg mice (E, I), in contrast to 3×Tg-AD mice (G, L). SBDP120 labeling was clearly present in the cortex of 24 month-old non-Tg (F, J) mice, but was dramatically increased in 3×Tg-AD (M) mice. The labeled elements were granular but frequently appeared to be arranged around cell borders in 24 month-old non-Tg and 12 month-old 3×Tg-AD cortex (J, L). In 24 month-old transgenic mice the labeling was aggregated around the cell perimeter as well as being present in the somata of pyramidal cortical neurons (M). Panel N is a psudocolor image with a background cutoff illustrating the age and genotype differences in relative density of SBDP120 reactivity in cortical layer V. These differences are expressed quantitatively in the bar graph (O). *p<0.005. Scale bar = 1 mm in A applying to B-D; equal to 200 µm for E–H and 50 µm for I–M.

**Figure 4:**
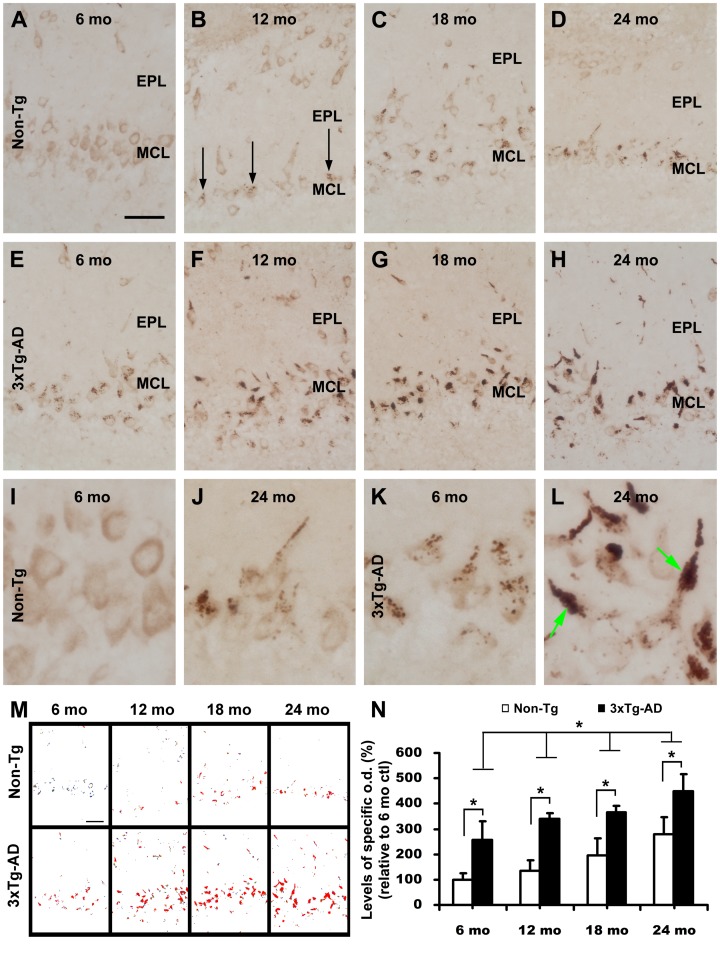
Images and densitometry showing increased SBDP120 immunolabeling in the olfactory bulb of 3 ×**Tg-AD mice relative to non-transgenic (Non-Tg) cohorts from 6 to 24 month of age.** Panels A-H illustrate the evolution of immunolabeling pattern around the mitral cell layer (MCL). In non-Tg mice, granules of strong SBDP120 immunoreactivity appeared in mitral cell somata at 12 months of age (B, arrows) and progressively increased at older ages (C, D, J); such granular elements were no seen in 6-month old non-Tg mice (A, I). In contrast, in the 3×Tg-AD mice labeled granules were present in most mitral cells at 6 month of age (E), and their number and labeling intensity increased in older animals (F-H). There was a progressive aggregation of the labeled granules in the proximal area of the apical dendrites with age (F-H, K, and L). Psudocolor images (M) show an overview of the age and genotype differences in relative density of SBDP120 reactivity. The age-related increase in labeling density measured over the mitral cell and external plexiform layer is quantified in panel N. *p<0.005. Scale bar = 200 µm in A applying to B-H; equivalent to 25 µm for I-L.

Overall, an age-related increase of localized SBDP120 immunolabeling was observed in both 3×Tg-AD and non-Tg mouse forebrain, but the increase was more prominent in the former (Figs. 3 and 4). Very few SBDP120 immunoreactive elements were seen in non-Tg mouse cortex until 12 months of age (Fig. 3A, E, I). However, many labeled elements were present in layers V and VI, and to a lesser extent in layers II and III, in 24-month old non-transgenics (Fig. 3B, F, J). In the 3×Tg-AD mice, some SBDP120 expressing elements were detectable in the cortex as early as 6 months of age (not shown). The immunoreactive elements were consistently detected in deep cortical layers (IV–VI) in 12-month old transgenics (Fig. 3C, G, L). These further increased by 18 months (not shown), and became abundant by 24 months of age (Fig. 3D, H, M). At high magnification the labeling appeared largely granular, often grouped seemingly along the peripheral border of cortical cells at younger age points (Fig. 3J, L). However, the labeling was also clearly present in the somata of a subpopulation of pyramidal neurons in 24-month old transgenics (Fig. 3M).

The development of intracellular aggregation of the labeled granules in cortical pyramidal neurons could be appreciated by comparing the labeling pattern between ages as well as genotypes (Fig. 3I–M). Age and genotype differences in labeling pattern and intensity could be easily seen in pseudocolor images subjected to a background cutoff at the level of non-specific labeling (obtained from sections that were processed in the absence of primary antibody; Fig. 3N). Densitometric analysis confirmed that the specific optic density of SBDP120 IR was significantly higher in the transgenics relative to non-Tg groups at 12 (p<0.05, Bonferroni posttests, same test below) and 24 months (p<0.05) of age (Fig. 3O). There were also statistically significant differences in specific optic density between 12 and 24 months of age for both the transgenics (p<0.05) and non-transgenics (p<0.001, one-way ANOVA, F = 38.8, df = 3, 12).

Distinct age-related changes in SBDP120 IR were seen in the olfactory bulb, mostly impressive around the MCL (Fig. 4). Most mitral cells, and likely the tufted cells as well (sparsely distributed large cells in the EPL and between glomeruli), expressed faint and diffuse SBDP IR in both the non-Tg and 3×Tg-AD bulbs (Fig. 4A–L). In addition to this light labeling, a few mitral cells in non-Tg brains contained strong immunoreactive granular elements at 12 months of age (Fig. 4B, arrows). These elements were increasingly seen in mitral cells at 18 and 24 months in both the cell body (often around the peripheral pole) and the proximal apical dendrites (Fig. 4C, D, J). In 3×Tg-AD mice, SBDP120 immunoreactive granules were already present in many mitral cells by 6 months of age (Fig. 4E, K). These elements tended to progressively accumulate and condense in the somata and proximal dendrites of the mitral cells from 12 to 24 months of age (Fig. 4F–H, L). As a result, most mitral cells contained heavily immunoreactive granules that accumulated at the apical dendrites in the 24 month-old 3×Tg-AD mice which, in some cases, appeared to cause local swelling or otherwise disconnection of the dendrites (Fig. 4H, L). Individually labeled dendrite-like processes or segments were also seen in increasing numbers with age in the EPL and around glomeruli (Fig. 4F–H).

The age-related increase in SBDP120 immunolabeling in the olfactory bulb in 3×Tg-AD mice relative to non-Tg counterparts was clearly visible in thresholded pseudocolor images of the region including the MCL and EPL (see Fig. 4M). For quantitative comparions, the data were normalized to the mean (defined as 100%) of the 6-month old control group. Overall, levels of SBDP120 IR increased with age in both the non-Tg (p = 0.0027, F = 8.48, df = 3, 12, one-way ANOVA) and 3×Tg-AD groups (p = 0.0024, F = 8.76, df = 3, 12). One-way ANOVA analysis revealed statistically significant genotype differences (p<0.05 to p<0.001) at all 4 age points (p<0.0001, F = 19.66, df = 7, 24) (Fig. 4N).

### Western Analysis of SBDP120 Levels in 3×Tg-AD Relative to non-Tg Mice

**Figure 5:**
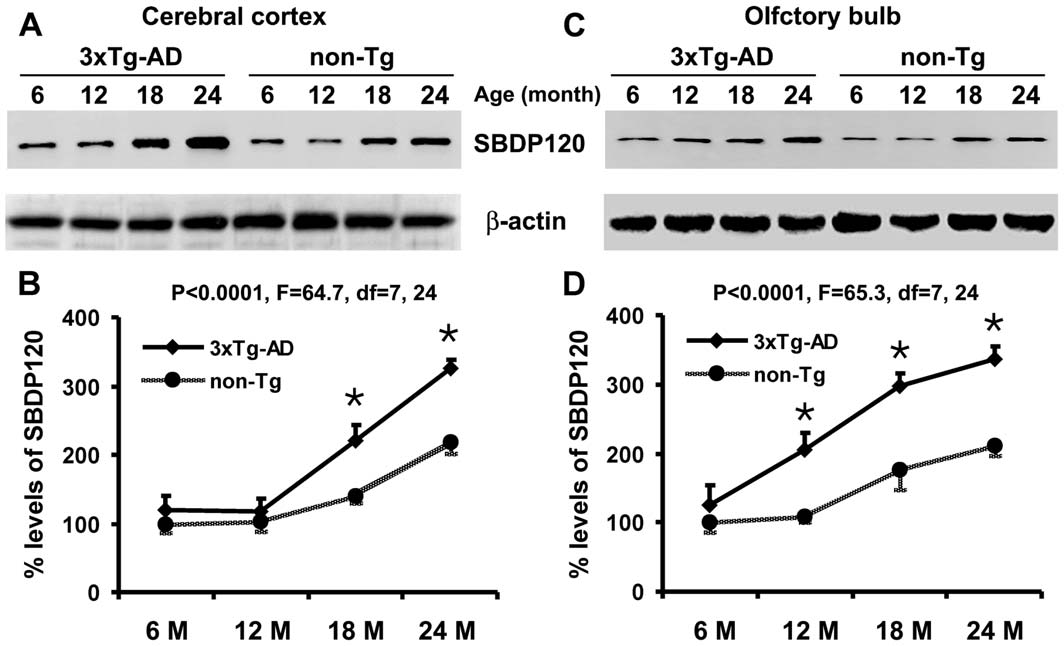
Age-related elevation of SBDP120 levels in the cerebral cortex and olfactory bulb of 3 ×**Tg-AD relative to non-transgenic mice.** Panels A and C show representative western blot results from one set of animals at the 4 age points as indicated. Graphs B and D show the quantitative data. Levels of SBDP120 were higher in transgenics relative to controls in the cortex at 18 and 24 months of age, and in the olfactory bulb from 12 months of age onward. N = 4 per data point. *p<0.001.

Western blot analyses were carried out to confirm the elevation of SBDP120 in the forebrain of the transgenic mice relative to non-Tg counterparts (n = 4 per point for each age and genotype; Fig. 5). The standardized SBDP120 signal (% of β-actin level) from each brain was normalized to the mean (defined as 100%) of the 6 month-old non-Tg group. SBDP120 levels in the non-Tg frontal cortex were 100±13.7%, 102.9±15.0%, 139.4±8.5% and 218.1±17.9% (mean±S.D., same format below) at 6, 12, 18 and 24 months of age, respectively, showing an age-related increase (p<0.0001, F = 62.5, df = 3,12). SBDP120 levels in the frontal cortex of 3×Tg-AD mice were also elevated with age (120.0±20.5%, 118.3.3±17.9%, 220.6±22.1% and 324.9±33.6%) at the above time points (p<0.0001, F = 65.6, df = 3,12). Significant differences were also found between the transgenic and non-Tg groups at 18 (p<0.001) and 24 (p<0.001) months of age (Fig. 5A, B).

Normalized SBDP120 levels in the non-Tg olfactory bulb were 100±13.3%, 108.9±7.9%, 176.1±29.2 and 210.1±12.9 at 6, 12, 18 and 24 months of age, respectively. There was a statistically significant increase with age (p<0.0001, F = 36.2, df = 3,12). In the 3×Tg-AD mice, relative levels of SBDP120 in the olfactory bulb were 126.0±28.6%, 205.3±25.3%, 298.0±17.0 and 337.2±26.1 at comparable age points, respectively, and also increased age-dependently (p<0.0001, F = 59.2, df = 3,12). Significant differences between transgenic vs non-Tg groups were seen at 12, 18 and 24 months of age (p<0.001 for all points; Fig. 5C, D).

### 6E10, p-Tau and Active Caspase-3 Immunolabeling in 3×tg-AD Forebrain

**Figure 6:**
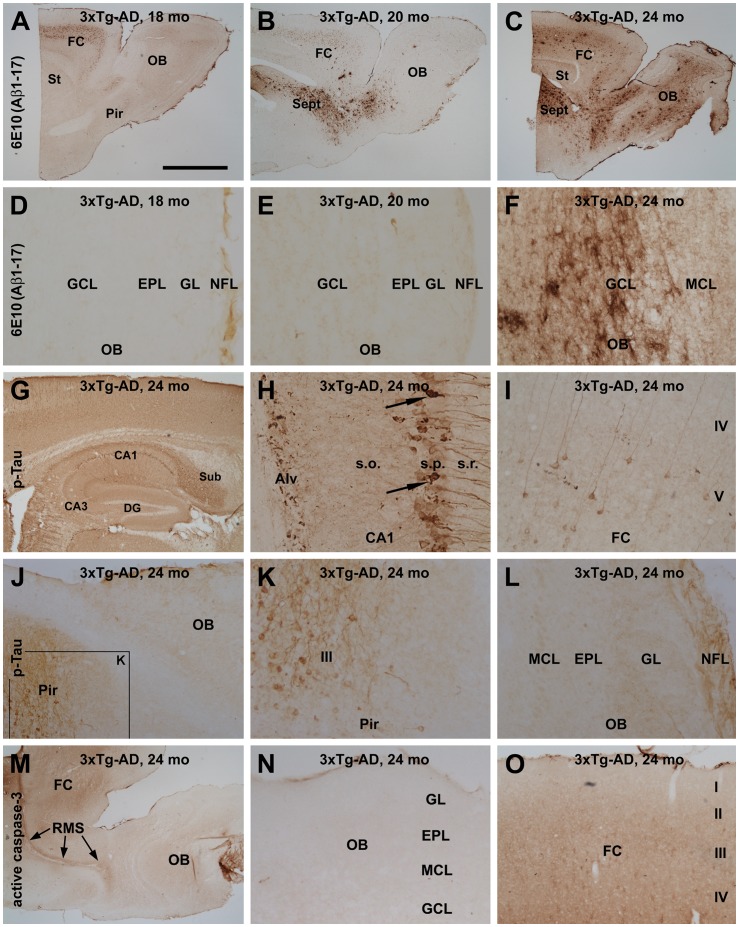
Images of immunolabeling with the β-amyloid (Aβ) antibody 6E10, phosphorylated tau (pTau) and active caspase-3 in the forebrain of 3 ×**Tg-AD mice.** 6E10 labeled cortical neurons in layers V and VI in the frontal cortex (FC) in 18- (A), 20- (B) and 24- (C) month old transgenics. Extracellular Aβ deposition appeared largely as diffuse plaques that were present in the cortex by 20 (B), but became common at 24 (C), months of age. Note the large number of plaques in the septum of both 20- and 24-month old transgenic mice. In the olfactory bulb, diffuse plaques appeared in the granule cell layer in 24 month-old animals (C, F); no 6E10 labeled neurons were seen in the olfactory bulb (OB) at any age point (D-F). Panels G-L show p-Tau labeling in the cortex, hippocampal formation and olfactory bulb in a 24-month old animal. Distinct labeling was seen in hippocampal CA1 pyramidal neurons and their processes, including axons in the alveus (Alv; G, H). A few CA1 pyramidal neurons exhibited tangle-like pathology (arrows in H). A small number of cortical pyramidal neurons also showed clear p-Tau immunoreactivity (I). p-Tau immunoreactive somata were not seen in the main olfactory bulb (J, L), but were present in the deep part of the piriform cortex (J, K). In contrast, p-Tau labeling was seen in the nerve fiber layer (NFL; L). No overt nuclear labeling of active caspase-3 was detectable in the forebrain in 24-month old animals (M-O). RMS: rostral migratory stream. Other abbreviations are defined in the legend for Figure 2. Scale bar = 2 mm in A applying to B-C, M; equal to 1 mm in G, 100 µm in F, and 200 µm for remaining panels.

The development of amyloid plaque and p-Tau pathology in the cerebral cortex and hippocampal formation in the 3×Tg-AD model has been described in many previous studies [39],[40],[42],[43],[44]. In our local colony of this mouse strain, the expression of the transgenic β-amyloid precursor protein (APP) in pyramidal neurons, as detected by 6E10, occurs in the cerebral cortex and hippocampus as early as 1–2 months of age, but extracellular amyloid plaques develop largely after 20 months of age in these forebrain areas [40]. In contrast, no 6E10-labeled neuronal somata labeling were seen in the main olfactory bulb in either young or old 3×Tg-AD mice (Fig. 6A–F), consistent with findings of a recent report [44]. A small amount of extracellular Aβ deposition could be detected in the olfactory bulb by 20 months of age in our colony of 3×Tg-AD mice (Fig. 6B, E). By 24 months of age, a considerable amount of extracellular 6E10 IR was present in the olfactory bulb (Fig. 6C, F). However, it should be stressed that this plaque pathology was present over the granule cell layer, but rarely around the outer bulb layers (the mitral cell, external plexiform and glomerular layers (Fig. 6C, F). Thus, there was no correlation (from regional, laminar or cellular distribution perspectives) between SBDP120 and 6E10 immunolabeling in 3×Tg-AD mice in the cerebral cortex or olfactory bulb.

p-Tau IR also appeared fairly early in our colony of the 3×Tg-AD mice, at 1–2 month of age. p-Tau labeling was most prominent in the subiculum and CA1, expressed in the somata and processes of principal neurons. A small population of cortical pyramidal neurons also expressed p-Tau IR in their somata and dendrites [40]. This overall regional and cellular pattern of p-Tau expression was maintained at older ages (after plaque onset), as shown in Fig. 6G–I. It should be noted that some hippocampal and cortical pyramidal neurons showed aggregated p-Tau reactivity resembling tangles (Fig. 6H, arrows). p-Tau immunoreactive neurons and processes were also present in layer III of the piriform cortex in young (not shown) and old transgenic mice (Fig. 6J, K). However, in the same immunostained section, no somal profiles were detectable in the olfactory bulb at any age [40], although weak IR existed in the nerve fiber layer (Fig. 6L). Therefore, as with 6E10, there was no apparent correlation between SBDP120 and p-Tau with respect to regional, laminar or cellular expression pattern at a given age in the 3×Tg-AD brain.

We also explored the anatomical correlation between SBDP120 and active caspase-3 labeling, using an antibody that labels active caspase fragments in cell nuclei [41]. Immunolabeling for active caspase-3 was carried out in both wild-type and transgenic mouse forebrain at various ages. These experiments did not reveal significant typical nuclear labeling of active caspase-3 in the cerebral cortex or olfactory bulb (Fig. 6M–O, shown as examples from a 24-month old mouse). Thus, laminar and cellular expression of SBDP120 IR did not appear to correlate with an overt/typical pattern of caspase-3 activation.

## Discussion

### SBDP120 Elevates in Aged Rodent Forebrain, and More Robustly in 3×Tg-AD Mice

Previous studies show increased levels of αII-spectrin breakdown in rodent brain during aging [31],[33],[34]. We attempted to determine the cellular/neuronal localization and time course of age-related SBDP elevation. We tested several rabbit and goat antibodies to various SBDPs; many showed diffuse cellular labeling in young rodent brain (data not shown), presumably because of a cross-reactivity of the antibody to uncleaved αII-spectrin [27]. The rabbit antibody to SBDP120 exhibited selective labeling. After a vigorous validation of its specificity, this antibody was considered suitable for use in immunohistochemical studies.

We demonstrate here that lamina and cell-specific SBDP120 labeling was present in the cortex and olfactory bulb in 16 month-old rats. In 24–26 month-old rats, specific SBDP120 labeling was prominent in the cortex and olfactory bulb. SBDP120 IR could be visualized in relatively large-sized layer V perikarya and some dendrite-like processes in aged rat cortex. Heavily labeled elements were also consistently present around the mitral cell layer in the olfactory bulb in aged rats. In line with these rat data, levels and immunolabeling density of SBDP120 increased progressively with age in both wild type and 3×TG-AD mouse frontal cortex and olfactory bulb. Protein levels and immunolabeling density of SBDP120 in these forebrain areas were comparatively higher in 3×Tg-AD mice than in their non-Tg cohorts. Together, these data suggest that the 120 kDa αII-spectrin breakdown product is upregulated in the forebrain with age, and that this phenomenon is enhanced in a transgenic model of AD.

### Olfactory Mitral Cells are Prone to SBDP120 Accumulation in their Apical Dendrites

The present study identifies that the olfactory bulb principal neurons are especially vulnerable to an early-onset but aggressive SBDP120 accumulation. Granular SBDP120 labeling appeared first around the junction of the soma and apical dendrite, by 12 months of age in non-Tg mice but at least as early as 6 months of age in 3×Tg-AD animals. With age, these granules continued to accumulate, largely in the apical dendrites. In old animals, SBDP120 immunolabeled granules sometimes filled in the entire apical dendrite of a mitral cell and seemed to cause local swelling in some cases. To our knowledge, this type of age-related mitral cell lesion has not been previously documented in literature. A potential reason for this exceptional SBDP accumulation might be that the mitral cell dendrites are postsynaptic to a very plastic presynaptic system, olfactory nerve terminals that undergo constant renewal [45]. One may expect that there is a continuing process of dynamic membrane remodeling on mitral cell dendrites due to the high plasticity of the first olfactory synaptic relay. Such a process requires a high rate of spectrin assembly and disassembly in the dendrites, which could lead to a buildup of SBDPs [16]. Other possibilities may include that the mitral cells have a less efficient calcium buffering system and/or autophagy-lysosomal clearance system. Of note, the mitral cells in human are vulnerable to age-related neurodegeneration, which is exaggerated in AD [46],[47]. Future studies will be needed to determine if there is a temporal/causal relationship between SBDP120 elevation and mitral cell death.

### Mechanistic Considerations for Intraneuronal SBDP120 Buildup

The molecular mechanism underlying age-dependent intraneuronal SBDP120 accumulation in normal rodents and 3×Tg-AD mice is currently unknown. SBDP120 is derived from caspases-3-mediated αII-spectrin cleavage, as has been shown in conditions promoting apoptotic and necrotic cell death conditions [19]. In the present study we did not detect a significant amount of nuclear labeling of active caspase-3 in the cortex or olfactory bulb. It should be noted that pharmacological studies indicate that caspase-3 activation is involved in many physiological cellular events under normal conditions, including modulation of long-term potentiation and other forms of synaptic plasticity, which are not associated with an overt presence of active caspase-3 fragments [48],[49],[50]. In addition, a degree of nuclear labeling of active caspase-3 can be seen in apparently healthy neurons in some brain regions [41],[48],[49],[51]. We hypothesize that a low dose of non-apoptotic caspase-3 activation may exist in vulnerable neuronal populations such as cortical and olfactory projective neurons [52], leading to the observed intraneuronal SBDP120 elevation. This level caspase-3 activation is below the threshold of detection by antibodies and may, perhaps, be involved in modulation of neuroplasticity rather than apoptosis. Such a notion is consistent with the finding that SBDP120 accumulation progresses over an extended period, given the initial appearance of labeling before mid-age.

Why do 3×Tg-AD mice show earlier intraneuronal SBDP120 labeling relative to non-Tg cohorts? This genotype-related difference does not appear to be *directly* caused by amyloid or tau pathology. In the cerebral cortex, SBDP120 labeling does emerge initially in the deep layers that exhibit prominent transgenic APP expression (based on 6E10 cellular labeling) [40],[42],[43]. Conversely, the olfactory mitral cells do not exhibit impressive 6E10 or p-Tau labeling in either young or aged transgenic mice [44]. Thus, other molecular/cellular disturbances in the transgenic brain must be responsible for the accelerated αII-spectrin breakdown. Among the potential candidates, oxidative stress [53],[54] or the calcium buffering deficits inherent in transgenic AD animal models are of particular interest [55]. Specifically, mutations in presenilins have been associated with calcium leakage and deregulation. This chronic calcium stress might activate calcium-sensitive proteases such as calpain and caspases to levels triggering spectrin degradation [56],[57],[58].

In summary, the present study identifies an age-related occurrence of the αII-spectrin cleavage product SBDP120 in forebrain neurons in normal laboratory rodents, which is accelerated in the triple transgenic model of AD. The olfactory bulb mitral cells are particularly vulnerable to this age-dependent neuronal change. The results suggest that the metabolism of a key membrane protein can manifest as an early neuronal alteration during brain aging and under chronic neurodegenerative conditions, perhaps as the consequence of dysfunctional calcium-sensitive protease signaling.
